# Absence of Asymptomatic Malaria Infection in Endemic Area of Bashagard District, Hormozgan Province, Iran

**Published:** 2012

**Authors:** H Turki, S Zoghi, A A Mehrizi, S Zakeri, A Raeisi, H Khazan, AA Haghdoost

**Affiliations:** 1 Malaria and Vector Research Group (MVRG), Biotechnology Research Center (BRC), Pasteur Institute of Iran, Tehran, Iran; 2 Department of Parasitology and Mycology, School of Medicine, Shahid Beheshti University of Medical Sciences, Tehran, Iran; 3 National Programme Manager for Malaria Control, Ministry of Health and Medical Education, Tehran, Iran; 4 Departement of Medical Entomology & Vector Control, School of Public Health, Tehran University of Medical Sciences, Tehran, Iran; 5 School of Public Health, Kerman University of Medical Science, Kerman, Iran

**Keywords:** *Plasmodium vivax*, *Plasmodium falciparum*, *Asymptomatic malaria*, *MSP-1*_*19*_, *Iran*

## Abstract

**Background:**

A successful malaria elimination program calls for enough attention to parasite carriers, especially asymptomatic malaria, as well as the diagnosis and treatment of clinical cases. Asymptomatic malaria is an infection that patients do not show any symptom; thus, these patients play critical role in the concept of an elimination program. The current investigation was conducted to evaluate the presence of these cases in Bashagard District, formerly a high malaria transmission area in Hormozgan Province, Iran.

**Methods:**

Blood samples (n = 500) were collected from symptomless individuals residing in Bashagard to evaluate *Plasmodium* infection by using microscopic, serological and nested-PCR techniques.

**Results:**

Regarding the microscopic and nested-PCR analysis, no asymptomatic infection was detected among studied individuals. Totally, 1% of the studied population (5 of 500) had anti PvMSP-1_19_-specific IgG antibody; however, only 0.2% (1 of 500) of the individuals was seropositive to recombinant PfMSP-1_19_, using ELISA.

**Conclusion:**

This study showed no asymptomatic malaria infection in the studied population; hence malaria elimination is feasible and can be successfully carried out in this region.

## Introduction

Malaria is the most important vector-born and parasitic disease and half of the world's population is under the risk of acquiring malaria (1). This infection is still the most important mosquito-borne disease in Iran and one of the main health problems especially in the south and southeast regions including Sistan and Baluchistan, Hormozgan and Kerman Provinces. The Iranian Center for Disease Management and Control (CDMC) reported 3,031 malaria cases in year 2010, of which 31.47% were imported cases from neighboring countries. Moreover, by using microscopy technique, it was understood that, 86.12%, 11.18% and 2.7% of the reported cases had been infected by *Plasmodium vivax*, *P. falciparum* mono-infection and a mixed-infection of both species, respectively (CDMC, unpublished data).

The Iranian CDMC with the technical support from WHO started the malaria elimination program in Iran since 2009. It should be noted that to achieve successful malaria elimination in any given endemic region, one of the main requirements is active case detection (2). Therefore, an assessment of the epidemiological characteristics of malaria infections in a certain population, particularly the prevalence and distribution of asymptomatic infections will contribute to the understanding of the requirements of diagnostics in malaria elimination.

To manage the risk of asymptomatic malaria infection in different endemic areas of the world, various studies evaluated the presence and prevalence of this infection in the control and elimination phase of malaria using different techniques (3–14). Malaria diagnosis is achieved by the microscopic examination of blood smears and it is able to detect parasite species and densities. In addition, PCR is a more sensitive technique than microscopy, and has been widely used for the confirmation of the diagnosis of malaria infections (6, 15). It should be considered that in most malaria control areas, where the goal is to reduce malaria morbidity and mortality, quality assured microscopy has played a crucial role to detect parasites in the majority of clinically suspected patients and thus can lead to a correct treatment. In contrast, for malaria elimination settings, it is essential to detect all sources of infection, including those with low, sub-microscopic parasitaemia as well as the asymptomatic carriers. In fact, these carriers act as parasite reservoirs in the population (16, 17) and continuously transmit parasite to the anopheline mosquitoes (18).

There are several reports of high prevalence asymptomatic infection from malaria endemic areas of Africa, Asia and South America including Nigeria (5), Senegal (6), Gabon (7), Yemen (8), Thailand (9), Burma (10), Amazon region of Brazil (11) and Peru (12). In contrast, in countries such as Sri Lanka (13) and Kenya (14) no asymptomatic malaria cases have been reported. Therefore, the challenges of malaria elimination in different areas vary widely.

As shown by different studies (3–14) malaria epidemiology varies between countries and regions due to the difference in mosquito vector species, parasites species, human populations, rainfall, temperature, housing conditions and population movement. As a result, each endemic area needs to investigate the malaria epidemiology and carefully adapt its case detection strategy to the local situation. As mentioned, the malaria elimination program started in 2009 in Iran; therefore, it is wise to assess the epidemiological characteristics of malaria infections, particularly the prevalence and distribution of asymptomatic infections by increasing the quality of malaria case detection, which is one of the most important requirements in malaria elimination.

To achieve this aim, this study was designed to evaluate the presence of asymptomatic infection in the malaria endemic region of Bashagard for the first time by using microscopy, serological and nested-PCR techniques. The consequence of this study can have an important practical implication to the malaria elimination strategy in Iran.

## Materials and Methods

### Study area

**Fig. 1:**
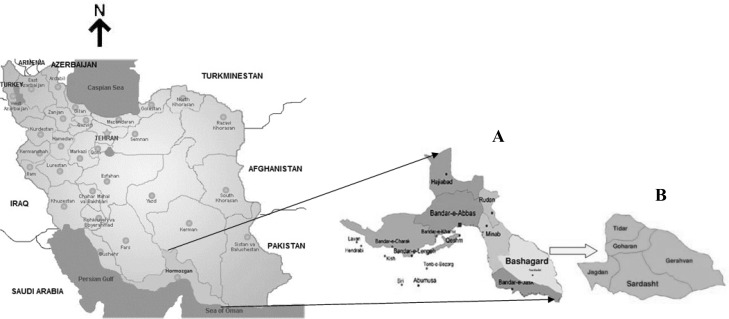
The map of Iran showing the study area; Hormozgan Province (A). Bashagard District (B)

This cross-sectional study was carried out in Bashagard area, Hormozgan Province, Iran. Bashagard is located in the Southeast of Hormozgan Province (Fig. 1). It is a large tropical mountainous area with an annual average temperature of 26 °C (7.7 – 44.2 °C), annual pluviometer index of 11.6 mm and an annual relative humidity of 46.2%. Bashagard has been considered to have the most reported malaria cases comparing to other districts of Hormozgan in the last ten years in accordance with the report of Provincial Health Center and CDMC of Iran (unpublished data). Malaria is seasonal with two peaks in Bashagard, the first peak is in May and the second in October. The malaria prevalence in this studied area is mainly due to *P. vivax* (98.5%) and, *P. falciparum* (1.5%) is less prevalent (CDMC of Iran, unpublished data). The most important anopheles vectors in Bashagard are *Anopheles stephensi*, *An. culicifacies* and *An. fluviatilis* (19). According to the report of the Iranian CDMC, the annual parasite incidence (API) in the studied areas was 0.4 in 2009, before moving toward malaria elimination strategies.

### Study population and sample collection

This study was conducted in 500 volunteers (64.2% female and 35.8% male), aged from 4 to 60 years old, living in Bashagard District. All randomly selected participants were found actively without having any malaria symptoms including chill, fever and sweating in order to evaluate asymptomatic *Plasmodium* infection in August 2009 (between the two seasonal peaks of malaria) in Bashagard District. Pregnant women and children below four years old were excluded from the study. After signing an informed consent form, finger-prick blood, thick and thin blood smears, were prepared from all cases for microscopy. But, for nested-PCR, and serological analysis, blood samples were collected in tubes containing EDTA. Plasma was obtained after the centrifugation of the collected blood samples and stored at -20 °C until use. If the positive result would be detected by microscopic examination, in this case the patient will be received standard anti-malarial drugs in accordance with CDMC of Iran. This study was approved by the Ethical Committee of Pasteur Institute of Iran.

### Blood smear examination for malaria parasites

Thick and thin blood smears were prepared according to a standard method. Briefly, after cleaning the finger with 70% ethanol it was punctured. Thick and thin blood smears were air-dried, and then only the thin film was fixed using methanol. All slides were stained with 10% Giemsa and examined at **×** 1000 with oil immersion for detecting malaria parasites by a trained microscopist at the Hormozgan Provincial Health Center laboratory.

### ELISA

IgG antibodies in serum samples were detected by ELISA using the 19 kDa C- terminal region of merozoite surface protein 1 (MSP-1_19_) of *P. vivax* and *P. falciparum* as recombinant antigens (20, 21). Briefly, ELISA was performed as follows: the plate was coated with rPvMSP-1_19_ (32 ng) or rPfMSP-1_19_ (200 ng) antigens per well overnight. Then the plate was blocked with the blocking buffer (1% BSA in PBS 1X) and washed three times with the washing buffer (1% PBS- 0.05% Tween-20). Afterward, 100 µL of sample sera was added to the wells (at 1:500 dilution for *P. vivax* and 1:200 dilution for *P. falciparum*) and incubated at room temperature for 1.5 h. Then the plate was washed and 100 µl of horse radish peroxidase-labeled anti-human IgG (diluted 1:30,000 in washing buffer; Sigma, USA) was added to each well. After 1 h of incubation the plate was washed and 100 µL orthophenylene diamine hydrochloride (OPD, Sigma, USA) substrate was added to each well. The reaction was stopped by adding 50 µl of 2 N H_2_SO_4_ and the absorbance (OD) was measured at 492 nm. It should be mentioned that all samples were analyzed in duplicate.

All IgG positive samples were also analyzed to determine the IgG1 and IgG3 subclasses. To measure the anti-PvMSP-1_19_ and –PfMSP-1_19_ IgG1 and IgG3 antibodies, the ELISA was carried out as described above but with Monoclonal Biotin conjugated anti Human IgG1 and IgG3 (Sigma, USA) as secondary antibodies at dilutions of 1:2000 and incubated at room temperature for 1 h. After washing, the procedure was followed by adding streptavidin–peroxidase conjugate (Sigma, USA) at a dilution of 1:3000 and incubated at room temperature for 1 h. The enzyme reaction was developed with OPD-H_2_O_2_ (OPD, Sigma, USA) and stopped with 2 N H_2_SO_4_. The samples with an optical density greater than the cut-off point were considered positive. The cut-off was calculated as the mean OD of the 30 samples collected from healthy individuals who are living in non malaria endemic region plus three standard deviations.

### Nested-PCR

Parasite genomic DNA was extracted from 300 µL of the blood samples using a promega kit according to the manufacturer's instruction (Promega, Madison, WI, USA). DNA samples were used to amplify the small sub-unit ribosomal ribonucleic acid (18ssRNA) gene with species specific primers for *P. vivax* and *P. falciparum* as described previously (22) with some minor modifications. Briefly, 5 µL of extracted DNA was added to the first reaction, using pairs of primers, targeting an outer region specific to the *Plasmodium* genus. Then 2 µL of the first nested-PCR product was used as a template in the second nested reaction to yield specific *P. vivax* and P. *falciparum* products. The annealing temperature was 72 °C for all reactions and the cycles repeated 25 and 30 times for the first and the second nested reaction, respectively. The nested-PCR products were electrophoresed on 2% agarose gel for *P. vivax* and *P. falciparum* and visualized under UV light after staining with ethidium bromide. A sample was considered positive for *P. vivax* and *P. falciparum* if a 120 and 205 base-pair product was detected, respectively. DDH2O and DNA of the predetermined *P. vivax* /*P. falciparum* infected samples were used as negative and positive controls, respectively.

## Results

To evaluate the presence of asymptomatic infection in Bashagard, 500 volunteers who did not have any malaria symptoms were selected. Sampling was performed during one week (3^rd^ to 10^th^ August 2009). All study samples were categorized by sex and age group (Table 1). The majority of samples were between the ages of 11-20 (38%) and 223 (44.6%) cases had a history of malaria infection during the past 10 years.

**Table 1:** Distribution of samples by age groups and gender

	Gender		

Age groups (yr)	Male No. (%)	Female No. (%)	Total No. (%)
4-10	36 (46.2)	42 (53.8)	78 (15.6)
11-20	69 (36.3)	121 (63.7)	190(38)
21-30	25 (33.8)	49 (66.2)	74(14.8)
31-40	21 (28.8)	52 (71.2)	73(14.6)
41-50	20 (39.2)	31 (60.8)	51(10.2)
51-60	9 (26.5)	25 (73.5)	34(6.8)
Total	180 (35.8)	320 (64.2)	500

### Microscopy

All 500 thick and thin Giemsa-stained blood smears were examined for detecting malaria parasite by an expert microscopist and no *Plasmodium* parasite was detected in any examined sample.

### Serological results

In the ELISA assay using rPvMSP-1_19_ as an antigen, 1% of the studied population (5 out of 500) had anti PvMSP-1_19_-specific IgG antibody to PvMSP-1_19_. Among them, 0.2 was under and 0.8% was ≥ 15 years old. Serological results using rPfMSP-1_19_ as an antigen showed anti PfMSP-1_19_-specific IgG antibody in only one of the samples (0.2%) who was a 35 years old woman (Table 2). In addition, IgG subclass analysis showed that IgG1 but not IgG3 subclasses were positive for all IgG positive samples (Table 2).

**Table 2:** Detection of anti-*Plasmodium* IgG antibodies (IgG, IgG1 and IgG3) to PvMSP-1_19_ (a) and PfMSP-1_19_ (b) recombinant proteins as antigens by using ELISA

a)	Sample code	Age (yr)	Optical density of anti-PvMSP-1_19_ antibodies		

			IgG	IgG_1_	IgG_3_
	1	15	0.77	0.404	-
	2	28	0.77	0.275	-
	3	18	1.4	1.043	-
	4	21	0.91	0.663	-
	5	12	0.79	0.301	-
	Cut-off		0.5	0.19	0.18

b)	Sample code	Age(year)	Optical density of anti-PfMSP-1_19_ antibodies		

			IgG	IgG_1_	IgG_3_
	1	35	1.8	0.527	0.133
	**Cut-off**		**0.53**	**0.16**	**0.18**

### Nested-PCR results

The nested-PCR technique was used to detect 18ssrRNA of *P. vivax* and *P. falciparum*. The nested-PCR by specific primers for specific fragments of *P. vivax* and *P. falciparum* was negative for all examined samples.

## Discussion

As the malaria elimination program started in 2009 in Iran, active case detection has become a crucial element during achievement. In this situation one of the main challenges is the detection of asymptomatic infection which is not detectable by routine laboratory tests due to the lack of malaria symptoms and having low levels of parasitaemia. So these patients become gametocyte carriers and have a key role in continuing disease transmission that might combat the malaria elimination program (1, 4, 11). Considering the challenge of asymptomatic malaria in the elimination program, the aim of this study was to evaluate the presence of asymptomatic malaria in Bashagard District, in Hormozgan Province which is one of the main malaria endemic areas of Iran. In order to increase the accuracy and reliability of the results, microscopic, serological and molecular techniques were simultaneously used to detect *Plasmodium* parasite among studied cases (as asymptomatic carrier) in this survey.

Microscopic technique was used as a gold standard and nested-PCR as a sensitive and specific method for asymptomatic malaria diagnosis which can detect low parasite densities (23). Despite the efficacy of microscopy in case of high parasitaemia (50-200 parasite/µl), more sensitive diagnostic tools such as nested-PCR (1-5 parasite/µl) are required for the diagnosis of low parasitaemic carriers in elimination phase (24). While serology may not be a conclusive method for patent malaria diagnosis, the presence of IgG antibodies against MSP-1_19_ have been shown to correlate with recent infection (1).

The results of this study did not indicate any asymptomatic infection neither by microscopic nor nested-PCR examinations in the studied population. However, in the case of serological examinations, five and one cases were positive and had anti-PvMSP-1_19_ and -PfMSP-1_19_ IgG antibodies, respectively. The present serologic data is parallel with the previous study (1), which is not as convincing as nested-PCR in the diagnosis of either patent or asymptomatic malaria infection.

The emerging of asymptomatic malaria infection could be a result of the repeated exposure of individuals to the parasite population which leads to the development of effective humoral immune response. Thus despite the presence of parasite, malaria symptoms might not be observed (2). Interestingly, in the current study, there was no asymptomatic infection in Bashagard District. This could be explained by the absence of a repeated exposure of individuals to the infectious pathogen; as a result, the immune response is not strong enough to initiate a symptomless infection due to the lack of sufficient boost. In addition, another reason for the absence of asymptomatic infection could be the successful administration of the malaria control program, particularly active case detection, in Bashagard District in recent years.

Although it is expected that asymptomatic infection would mainly be detectable in high endemic areas, there has been other contrary reports from hypoendemic areas of the world that indicated high prevalence of asymptomatic malaria such as Peru and Brazil (1, 4, 12). The inconsistency between those results with the current results from Bashagard District, in spite of similar malaria endemicity, could be explained by the difference in host and/or parasite genetics characteristics.

Taken together, the results of the present survey showed that there was no asymptomatic malaria infection in the studied population of Bashagard District. Therefore, it could be postulated that, as no asymptomatic malaria cases were found, the malaria elimination program is feasible and can be successfully carried out in this area. As reported previously from Iranshahr District of Sistan and Baluchistan Province, asymptomatic infection was detected by microscopy among both Iranian individuals and Afghani immigrants (25). Therefore, to achieve elimination in all over the country, further studies are required in this malaria endemic province as a highly endemic setting with more imported cases from Eastern neighboring countries.
